# The Causation of Disease
*"An Exposition of the Ultimate Factors which induce it." By Harry Campbell, M.D., B.S., London, Member of the Royal College of Physicians; Assistant-Physician and Pathologist to the North-West London Hospital. H. K. Lewis, 136, Gower-street. 1889.


**Published:** 1889-05-04

**Authors:** 


### The Causation of Disease.*

The object of this very learned work is to trace out the ultimate causes of disease—not of particular diseases, but of disease in general. Dr. Campbell's opening chapter consists of "General Remarks on Causation," it being necessary at the outset to attach a definite meaning to the word "cause." This, however, has been attempted before: Whewell, Hume, Locke, Bacon, Kant, Brown, and others have written on the subject. In Grove's "Correlation of the Forces" we find the following passage, written nearly 20 years ago: "Although the word cause may be used in a secondary and concrete sense, as meaning antecedent forces, yet in an abstract sense it is totally inapplicable." Dr. Campbell defines the "law of causation" thus: "Given certain materials, the same results will always follow"; and he defines the cause of any particular effect as the sum of those material conditions from which that effect has necessarily followed. But this definition assumes the existence of a material world, and the author states it is utterly impossible to prove the existence of such material world, since we have no knowledge of anything but our own mental states. This is, perhaps, theoretically correct, and is in accordance with Locke, whose denial of the existence of substance will be recollected; but it trenches too much on mystic Hindu philosophy for acceptation by practical minds. The Vedanta school of Hinduism denies the reality of the visible world, and calls it "maya," or "delusion." The Hindu philosophers say the visible universe is a projection of the spirits, as a shadow in the magic-lantern. They attribute it to two effects—viz.: enveloping the soul, which gives rise to the conceit of personality, and projecting the appearance of the world, which the individual imagines to be external to himself. It is, in fact, an attempt by the Hindus to explain the consciousness of man and the existence of an external world, in accordance with the sole existence of God and the principle *ex nihilo, nihil fit*. One of the authors mentioned above (Whewell) states: "Things are something different from ourselves and independent of us, something which is without us; they are; we see them, touch them, and thus know that they exist." With this we fully agree, and are glad to find that although Dr. Campbell says it is utterly impossible to prove the existence of a material world, he nevertheless agrees that we are at perfect liberty to assume so. If there were no material world, if all were *maya* or illusion, or if we were not at liberty to assume the existence of a material world, Dr. Campbell's book would not have been written, for it deals with material things. Minuteness of cell life is of no consequence. The author tells us that the atomic particles of a protoplasmic cell may be as small in comparison with the cell, as is this latter in comparison with the earth. Still, if it is matter, it must be material, and therefore subject to laws. Now, most writers on the point opine that the idea of causation cannot be separated from that of force. It has been argued that force is that which produces and resists motion. Heat produces motion; *ergo*, heat is the cause of motion. On the other hand, motion produces heat; so motion is the cause of heat. But this does not land us any nearer an ultimate cause. Neither does the conception of force by Herbert Spencer, quoted by the author, to the effect that it is the mental ingredient out of which our conceptions of matter, space, time, and motion are built. We certainly do not think Dr. Campbell's first chapter assists us much in following the after articles on the causation of disease. It treats of questions the like of which have perplexed theologians, philosophers, and metaphysicians in every age, and will perplex them till the end of time. We know no more, for instance, of the cause of gravitation than we know of the cause of the universe. Chemistry may resolve the fabric of the world into elements, but the question still remains, where did those elements come from? We are told by Sextus that Protagoras was condemned to death because he confessed himself unable to say whether the gods existed, or what they were, owing to the insufficiency of knowledge. The expectation of arriving at ultimate cause or essences has continued from the earliest times. Although human pride is skilful to invent, to hide its ignorance, there are more things in heaven and earth than dreamt of in our philosophy. The tendency of all discovery has led to the conviction that all the affairs of the universe are under the regulation of laws. But the laws display intelligence, and the expression of will and power, beyond nature up to nature's God. Why, therefore, talk, why argue, why waste breath in trying to prove the unknowable? As regards ultimate cause, it may certainly be stated, thus far shalt thou go, but no farther. Science may object that by resting on assumption we remove from the domain of scientific enquiry. This, unfortunately, cannot be helped. It is not permitted to the finite to penetrate the infinite, and the pursuit of science is but the seeking of a deeper acquaintance with the infinite, which beyond a certain point has doubtless been forbidden.

Having taken the only possible course open, and referred effect to material conditions, Dr. Campbell defines disease to be an abnormal mode of life in such conditions, but unfortunately drifts off into a disquisition as to "how we shall define life, and what shall we say is the cause of it?" The answer given by the author is as follows: "A simple cell consists of a peculiar grouping of certain kinds of atoms into molecules, and a peculiar grouping of the molecules among themselves, each grouping being such . . . . that a certain interaction takes place between the cell and the cell environment. This action is termed life." From this we venture to dissent entirely. That bodies are made up or composed of certain parts, elements, or principles, is a conception which has existed in men's minds from the beginning of the first attempts at speculative knowledge. The doctrine of the four elements—earth, air, fire, and water—of which all things in the universe were supposed to be constituted, existed among the ancient Egyptians; but life or vital principle has always been separated from material.

The Duke of Argyle remarks: "Let us never forget that life, as we know it here below, is the antecedent or cause of organisation and not its product, that the peculiar combinations of matter, which are the homes and abodes of life, are prepared and shaped under the control and guidance of that mysterious power we know as vitality." With the above we fully agree. Moreover, if life is an interaction between cell and cell environment, and disease an abnormal interaction or mode of life, it should be explained why from accident and injury, or even bad news, life suddenly ceases, without prior disease or abnormal mode of life. On this head we find it stated the cell environments may be morbidly modified by nervous influence and violence. These, however, are statements, not explanations. Neither do we know what nervous influence is. Nervous influence ceases with life, and may be the same as life. We really are unable to accept the following as any explanation of nervous influence: "The striking of a nervous influence on a cell constitutes a modification of environment, since the influence comes from without. In this way the muscle dynamite is exploded, and the zymogen of the gland converted into zyme, while the direct influence of the nervous system upon cellular elements is abundantly shown in the field of pathology." Yet we fully agree with the author that the nervous system plays an enormous *rôle* in the phenomena of disease, although, as we think, in a manner not yet explained.

* "An Exposition of the Ultimate Factors which induce it." By Harry Campbell, M.D., B.S., London, Member of the Royal College of Physicians; Assistant-Physician and Pathologist to the North-West London Hospital. H. K. Lewis, 136, Gower-street. 1889.

